# High stereographic resolution texture and residual stress evaluation using time-of-flight neutron diffraction

**DOI:** 10.1107/S1600576718004004

**Published:** 2018-05-09

**Authors:** Pingguang Xu, Stefanus Harjo, Mayumi Ojima, Hiroshi Suzuki, Takayoshi Ito, Wu Gong, Sven C. Vogel, Junya Inoue, Yo Tomota, Kazuya Aizawa, Koichi Akita

**Affiliations:** a Materials Sciences Research Center, Japan Atomic Energy Agency, Ibaraki 319-1195, Japan; b J-PARC Center, Japan Atomic Energy Agency, Ibaraki 319-1195, Japan; c Department of Materials Engineering, University of Tokyo, Tokyo 113-8656, Japan; d Neutron Science and Technology Center, Comprehensive Research Organization for Science and Society, Ibaraki 319-1106, Japan; e LANSCE, Los Alamos National Laboratory, Los Alamos, NM 87545, USA; f Research Center for Advanced Science and Technology, University of Tokyo, Tokyo 153-8904, Japan; g National Institute for Materials Science, Ibaraki 305-0047, Japan

**Keywords:** neutron diffraction, texture measurement, stress tensor analysis, multilayered steels, limestone

## Abstract

Neutron diffraction texture measurements provide bulk averaged textures with excellent grain orientation statistics, even for large-grained materials, owing to the probed volume being of the order of 1 cm^3^. Furthermore, crystallographic parameters and other valuable microstructure information such as phase fraction, coherent crystallite size, root-mean-square microstrain, macroscopic or intergranular strain and stress, *etc.* can be derived from neutron diffractograms. A procedure for combined high stereographic resolution texture and residual stress evaluation was established on the pulsed-neutron-source-based engineering materials diffractometer TAKUMI at the Materials and Life Science Experimental Facility of the Japan Proton Accelerator Research Center, through division of the neutron detector panel regions. Pole figure evaluation of a limestone standard sample with a well known texture suggested that the precision obtained for texture measurement is comparable to that of the established neutron beamlines utilized for texture measurement, such as the HIPPO diffractometer at the Los Alamos Neutron Science Center (New Mexico, USA) and the D20 angle-dispersive neutron diffractometer at the Institut Laue–Langevin (Grenoble, France). A high-strength martensite–austenite multilayered steel was employed for further verification of the reliability of simultaneous Rietveld analysis of multiphase textures and macro stress tensors. By using a texture-weighted geometric mean micromechanical (BulkPathGEO) model, a macro stress tensor analysis with a plane stress assumption showed a rolling direction–transverse direction (RD–TD) in-plane compressive stress (about −330 MPa) in the martensite layers and an RD–TD in-plane tensile stress (about 320 MPa) in the austenite layers. The phase stress partitioning was ascribed mainly to the additive effect of the volume expansion during martensite transformation and the linear contraction misfit between austenite layers and newly transformed martensite layers during the water quenching process.

## Abstract 2

The division of neutron detector panel regions has improved the precision of complex texture evaluation and appropriate sample rotation enhances the texture reliability in a limited neutron beam time. The TAKUMI instrument has achieved satisfactory texture precision for a limestone standard sample. The compressive rolling direction–transverse direction in-plane stress field was quantitatively measured in martensite layers of a martensite–austenite multilayered steel, and this stress field was found to originate from the martensite transformation strain and the linear contraction misfit between austenite layers and newly transformed martensite layers.

## 1. Introduction   

Neutron diffraction is widely used as an accurate probe for materials texture evaluation because its large spot size and high penetration compared with X-ray and electron diffraction enable the acquisition of orientation information from a polycrystalline bulk sample averaged over a volume of the order of 1 cm^3^. Crystallographic information, *e.g.* atomic positions, lattice parameters, phase fractions, and strains or stresses, can also be obtained simultaneously. The excellent reliability of neutron texture analysis was confirmed by a round-robin experiment (Wenk, 1991 ▸). Utilizing the high penetrability of neutrons into many structural materials, various sample environments can be constructed to carry out *in situ* texture evaluations (Wenk & Van Houtte, 2004 ▸).

For constant-wavelength (or angle-dispersive, AD) neutron diffraction (Bunge *et al.*, 1982 ▸; Xu *et al.*, 2008 ▸, 2012 ▸; Hansen *et al.*, 2008 ▸; Brokmeier *et al.*, 2011 ▸; Zhang *et al.*, 2011 ▸; Li *et al.*, 2016 ▸), only a few low-*Q* diffraction peaks can be collected simultaneously depending on the stereographic angular coverage of the single-tube, one-dimensional or two-dimensional position-sensitive detector (PSD) panel. Here, *Q* is the momentum transfer, *Q* = 2sinθ*K*_i_ = 4πsinθ/λ = 2π/*d*, where 2θ is the scattering angle, *K*_i_ is the incident momentum, λ is the wavelength of the incident neutron beam and *d* is the lattice plane spacing, usually abbreviated as the *d* spacing. Although the collecting time of neutron diffractograms is usually ≤60 s for each sample rotation using an Eulerian cradle, in total it takes about 10 ks or longer to measure enough pole figures through many sample rotations, especially for multiphase and/or low-crystal-symmetry materials. Since this method relies on the integrated diffraction intensities of well resolved peaks, pole figure measurements of low-crystal-symmetry or multiphase materials are difficult to realize with a single-tube detector or a narrow 2θ-spanned PSD. Consequently, the simultaneous measurement of as many diffraction peaks as possible using a wide 2θ-spanned one-dimensional PSD (Bunge *et al.*, 1982 ▸; Hansen *et al.*, 2008 ▸) and the utilization of full profile refinement (Rietveld, 1969 ▸) are very valuable, and new-generation neutron diffractometers with a wide 2θ-spanned two-dimensional PSD, *e.g.* WOMBAT at the Australian Nuclear Science and Technology Organization (Simons *et al.*, 2014 ▸), are attracting much attention for their potential rapid-response capability for time-sliced texture measurements.

By comparison, time-of-flight (TOF or energy-dispersive) neutron diffraction (Wenk *et al.*, 2003 ▸, 2012 ▸; Kockelmann *et al.*, 2006 ▸; Conrad *et al.*, 2008 ▸; Onuki *et al.*, 2016 ▸) is employed to collect a complete diffractogram spanning a wide *Q* range from typically 1.1 to 12.5 Å^−1^ (corresponding to a *d*-spacing range from 5.5 to 0.5 Å) from each neutron detector panel covering a relatively large stereographic angle. Thus, many high-*Q* diffraction peaks (more than 100 peaks for low-symmetry materials) are collected simultaneously with low-*Q* diffraction peaks. After appropriate correction of the peak intensities, *e.g.* for incident intensity normalization, structure factor refinement *etc.*, each TOF neutron diffractogram corresponds to an inverse pole figure for each constituent phase for the sample direction defined by the diffraction vector for the corresponding detector panel. Although the collecting time of a TOF neutron diffractogram is usually more than 60 s for each sample rotation, having sufficient detector panel coverage means that only a few rotations (usually needing less than 3.6 ks in total) are required to obtain enough data to determine the orientation distribution function (ODF). Since the new neutron sources recently commissioned at the Spallation Neutron Source, Oak Ridge National Laboratory, Tennessee, USA, and the Japan Proton Accelerator Research Center (J-PARC), Ibaraki, Japan, and those soon to be commissioned at the China Spallation Neutron Source, Dongguan, Guangdong, China, and the European Spallation Source (ESS), Lund, Sweden, are pulsed neutron sources using the TOF technique, the approach discussed here is expected to become the standard approach for texture analysis using TOF neutron diffraction. As the proton flux at spallation source facilities increases, the available neutron beam flux becomes even stronger, so the planned ESS accelerated proton flux of up to 5 MW is expected to provide better *in situ* texture and microstructure monitoring capabilities for advanced polycrystalline materials.

It should be mentioned that the high-energy X-raysof between 30 keV and 1 MeV provided by modern synchrotron radiation sources such as the European Synchrotron Radiation Facility (Grenoble, France) can measure local textures, strains and phases much faster than modern neutron sources because their beam flux is several orders of magnitude higher (Liss *et al.*, 2003 ▸). They are increasingly used to characterize the *in situ* textures of fine-grained materials under various environmental conditions, such as at high pressure and under anisotropic stress (Wenk *et al.*, 2014 ▸), as well as for the local texture profiling of specific engineering parts (Coelho *et al.*, 2010 ▸), but the very narrow X-ray beam (several tens of micrometres) may result in some difficulty in obtaining high-statistics bulk-averaged textures and other related information for coarse-grained or heterogeneous materials. Thus, complementary usage of synchrotron X-ray diffraction and neutron diffraction is very valuable for clarifying complex materials processing.

Besides the crystallographic orientation distribution, the micro lattice strains and stresses and the macro stress tensors of textured samples are also valuable parameters for the study of elastoplastic deformation behaviors (Suzuki *et al.*, 2012 ▸; Xu *et al.*, 2015 ▸). Diffraction methods allow the deconvolution of the macro strains (averaged over the whole sample), the strains in the constituent phases (allowing characterization of load partitioning) and the intergranular strains related to stress incompatibility between differently oriented grains (due to elastic anisotropy of the single crystals). Precise lattice strain measurements are helpful for understanding grain-to-grain interactions during and after elastoplastic deformation (Wang *et al.*, 2003 ▸; Wroński *et al.*, 2007 ▸), high-temperature creep-fatigue behavior (Mamun *et al.*, 2014 ▸) and recrystallization texture evolution (Wang *et al.*, 2003 ▸). Conventional stress analysis using X-ray diffraction methods usually uses the direct linear relationship of strain *versus* sin^2^ψ, where ψ is the angle between the normal direction (ND) of the sample and ND of the diffracting plane bisecting the incident and diffracted beams. Since such an analysis method is based on the assumption of the constituent phases being distributed uniformly with a random distribution of grain orientations (Krawitz, 2001 ▸; Wang *et al.*, 2003 ▸; Fitzpatrick *et al.*, 2005 ▸), it cannot ensure a reliable determination of the residual stress field for a strongly textured material with large intergranular stress because the strain *versus* sin^2^ψ relationship may become strongly nonlinear (Krawitz, 2001 ▸; Wang *et al.*, 2003 ▸; Fitzpatrick *et al.*, 2005 ▸). Recently, the technique of combined Rietveld texture and strain/stress analysis has been developed (Ferrari & Lutterotti, 1994 ▸; Lutterotti, 2010 ▸; Wenk *et al.*, 2014 ▸) through implementing the BulkPathGEO texture-weighted geometric mean micromechanical model (Matthies *et al.*, 2001 ▸; Wenk *et al.*, 2014 ▸) and the Popa–Balzar texture-weighted strain/stress orientation distribution function (WSODF) model (Popa & Balzar, 2001 ▸; Balzar *et al.*, 2010 ▸).

A high-precision texture measurement is thus an essential prerequisite to realize a satisfactory combined analysis of texture and stress. TOF powder diffractometers for general texture analysis, such as the HIPPO beamline at Los Alamos Neutron Science Center (LANSCE), New Mexico, USA (Wenk *et al.*, 2003 ▸), and iMATERIA at J-PARC (Onuki *et al.*, 2016 ▸), are not optimized for strain measurement, possibly resulting in a large uncertainty in strain/stress analysis (Wang *et al.*, 2002 ▸, 2003 ▸; Wenk *et al.*, 2010 ▸), for example, due to the short flight path length of 8.8 m for HIPPO, even with the available strong neutron flux (Wenk *et al.*, 2003 ▸). For comparison, engineering material neutron diffractometers with detectors of 2θ = 90° and long flight path lengths of >35 m, such as ENGIN-X (ISIS, Rutherford Appleton Laboratory, Didcot, UK), SMARTS (Los Alamos National Laboratory, New Mexico, USA), VULCAN (Oak Ridge National Laboratory) and TAKUMI (J-PARC), are thought necessary to realize reliable orientation-dependent strain/stress analysis. Since the bulk texture part of the combined Rietveld analysis does not rely on high instrumental resolution or a well aligned diffraction gauge volume in the center of the sample, Rietveld texture analysis may be conducted concurrently. Knowledge of the ODF is essential to calculate accurate stresses from measured strains (Wang *et al.*, 2002 ▸; Larsson *et al.*, 2004 ▸).

Here, a combined Rietveld bulk texture and residual stress evaluation procedure is established on the TOF neutron engineering materials diffractometer TAKUMI. The effect of panel-region division on the accuracy of the texture evaluation is first investigated by comparing the full pole figures obtained on TAKUMI for an experimentally deformed limestone sample with other results from several neutron facilities used in the last round robin. For example, this limestone standard sample was repeatedly measured on the GPPD neutron diffractometer (Argonne National Laboratory, Illinois, USA) and the HIPD neutron diffractometer (Los Alamos National Laboratory) to evaluate the reliability of the generalized spherical-harmonic function description in the *General Structure Analysis System* (*GSAS*) software through analyzing the pole figures (Von Dreele, 1997 ▸). This sample was then employed again on HIPPO to examine the capability and reliability of the texture measurement environment (Wenk *et al.*, 2003 ▸).

A cold-rolled and annealed interstitial-free (IF) steel and a martensite–austenite multilayered steel were then employed as reference samples for a combined high-precision texture and microstructure evaluation for textured engineering materials. The multiphase macro stress tensors of the multilayered steel were also analyzed using the BulkPathGEO texture-weighted geometric mean model (Matthies *et al.*, 2001 ▸), and the reliability of their analysis is discussed.

## 2. Experimental methods   

### 2.1. Sample preparation   

Three samples were employed in this study: an experimentally deformed limestone (CaCO_3_, sample No. K433) (Wenk, 1991 ▸; Wenk *et al.*, 2003 ▸) in the form of a cube 8.0 mm on a side with rounded corners and edges, an SUS301/SUS420J2 austenite–martensite multilayer steel sheet (Xu *et al.*, 2012 ▸), and a cold-rolled and fully annealed IF steel (Xu *et al.*, 2008 ▸). The limestone, with a trigonal crystal structure, results in more complex diffractograms than the steel samples and is currently being used in a second round-robin test, three decades after the first one, to determine the reliability of neutron pole figure measurement and analysis at modern neutron sources. The preparation details of the limestone standard sample have been reported elsewhere (Wenk, 1991 ▸; Wenk *et al.*, 2003 ▸).

**Figure 1:**
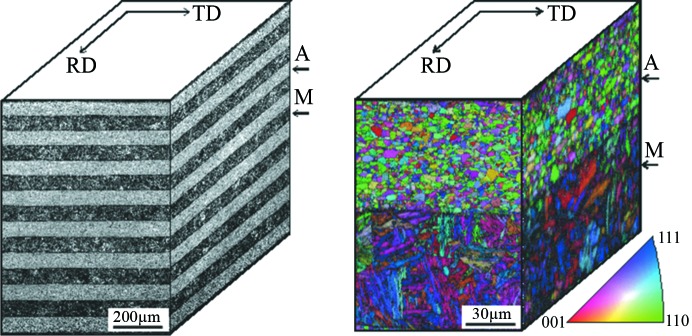
(*a*) Scanning electron microscope image of SUS301/SUS420J2 austenite–martensite multilayered steel along the thickness direction. Black shading denotes martensite (M) and gray shading denotes austenite (A). (*b*) Grain orientation map of the martensite–austenite layers obtained from EBSD. The various colors in the inset standard inverse pole figure (IPF) scale are used to denote the different grain orientations in the IPF mappings of martensite blocks and austenite grains by referring to the normal direction (ND) of multilayered steel; for example, the red grains have 〈001〉 || ND, the green grains have 〈110〉 || ND and the blue grains have 〈111〉 || ND. Grain boundaries are shown with a misorientation angle of 15°.

The multilayered steel sheet, composed of eight layers of austenite and seven layers of martensite, was employed to examine the reliability of the texture measurement by comparison with the previously published results obtained from the RESA-2 angle-dispersive neutron diffractometer with a 2θ = 7°-spanned one-dimensional PSD at the Japan Research Reactor No. 3 (JRR-3). The nominal chemical compositions of SUS301 and SUS402J2 (mass%) were 0.10 C–0.66 Si–0.97 Mn–7.02 Ni–17.02 Cr and 0.32 C–0.79 Si–0.60 Mn–13.52 Cr, respectively. These steels were multi-pass hot rolled at 1373–1273 K to reduce the thickness by 90%, with subsequent 50% cold rolling to obtain 1 mm thick steel sheets, followed by 1273 K rapid solution treatment for 120 s. The monolithic constituent steel sheets were isothermally annealed at 973 K for 3.6 ks. Cubic samples (10 × 10 × 10 mm) of these 1 mm thick steel sheets were prepared by spark cutting and then stacked together with cyano­acrylate adhesive as instant glue while preserving a common rolling direction (Xu *et al.*, 2012 ▸). The spark cutting avoids microstructure changes due to the temperature history, as the heat-affected zone is negligible after light grinding of the spark-cutting surface. The microstructures were observed with an Hitachi S-4300SE field-emission scanning electron microscope equipped with an electron backscatter diffraction (EBSD) system. Fig. 1 ▸ shows the scanning electron microscopy microstructure and EBSD grain orientation mapping of the multilayered steel in the rolling direction (RD) and transverse direction (TD) sectional planes.

The 0.68 mm thick cold-rolled and annealed IF steel was employed here as a macro-stress-free reference sample for the estimation of measurement error during the combined Rietveld texture and strain/stress analysis. The nominal chemical composition was 0.0018 C–0.01 Si–0.17 Mn–0.013 P–0.006 S–0.01 Cu–0.01 Ni–0.02 Cr–0.003 V–0.03 Ti–0.026 Nb–0.033 Al_solute_–0.0014 N_total_ (mass%) (Xu *et al.*, 2008 ▸). The 15 × 15 mm sheets were stacked up to form a cubic sample with consistent rolling direction, and the corners and edges were truncated to reduce the effect of geometric anisotropy of this cubic sample.

### 2.2. Neutron diffraction texture measurement   

**Figure 2:**
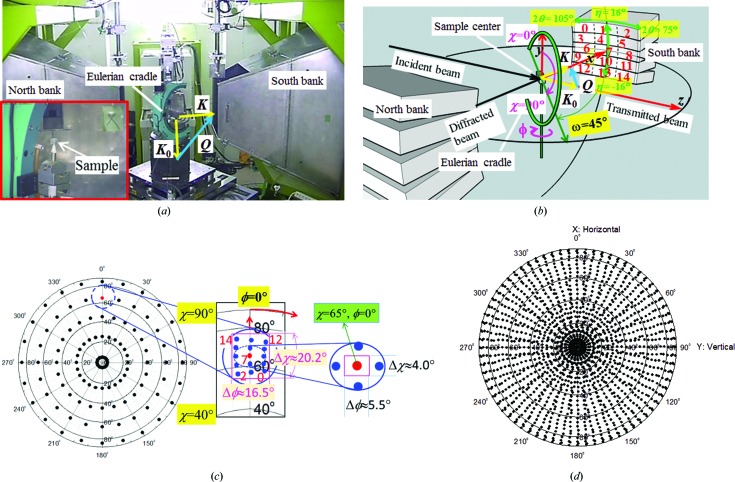
(*a*) A view of the geometric setting for simultaneous bulk texture and strain pole figure measurements on TAKUMI using an Eulerian cradle. (*b*) The definition of the instrumental *XYZ* coordinates, χ and ϕ sample rotation axes, panel-region division, and the corresponding numbering for the 2θ = 90° scattering detector bank. **K**_0_, **K** and **Q** are the incident, diffraction and scattering vectors, respectively. (*c*) The orientation coverage of 120 sample rotations for full pole figure measurement. The inset figure shows an enlarged example for the orientation coverage of all 15 central positions of panel regions at the sample orientation (χ = 65° and ϕ = 0°), and the red numbers 0, 2, 12, 14 and 7 mark the corresponding positions of the four corner panel regions and the central panel region in (*b*). (*d*) The orientation coverage of all the panel regions for full pole figure measurement.

TAKUMI (Moriai *et al.*, 2006 ▸; Harjo *et al.*, 2011 ▸) is an engineering materials TOF neutron diffractometer at the Materials and Life Science Experimental Facility (MLF), J-PARC (Takada *et al.*, 2017 ▸; Nakajima *et al.*, 2017 ▸; Sakasai *et al.*, 2017 ▸), dedicated to *in situ* microstructure and stress evaluation during hot (or warm or cryogenic) deformation and *ex situ* microstructure and residual stress measurements of large engineering components (Fig. 2 ▸ *a*). Its flight paths from the pulsed neutron source to the sample position and from the sample position to the 2θ = ±90° scattering detector banks are *L*_1_ = 40.0 m and *L*_2_ = 2.0 m, respectively. A 30.0 m long curved neutron guide is used in flight path *L*_1_ to reduce the number of unwanted fast neutrons and the amount of γ-radiation at the instrument. TAKUMI is installed at the high-flux neutron source facility viewing a poisoned decoupled hydrogen moderator with a typical neutron pulse shape profile. These conditions enable it to achieve the highest instrumental resolution at Δ*d*/*d* = Δ*Q*/*Q =* 1.7 × 10^−3^, where *d* is the lattice spacing. The south and north detector banks cover the same azimuthal angle range η = −16 to 16°, while their diffraction angle ranges are 2θ = 75 to 105° and −75 to −105°, respectively. TAKUMI uses ten scintillator-type one-dimensional detector panels consisting of 360 channels with a positional resolution of 3 mm (width) and 200 mm (height), which is equivalent to an angular resolution of about 0.086° (Δ2θ) × 5.73° (Δη). Five detector panels are stacked vertically in each detector bank, as shown in Fig. 2 ▸(*b*).

For texture measurements, 120 neighboring detector channels (2θ spanning ∼10°) are grouped into a neutron panel region, so that for each detector bank 15 panel regions (with centering diffraction angles of 2θ = 80, 90, 100° and centering azimuthal angles of η = ±11.46, ±5.73, 0°) are employed to collect neutron diffractograms simultaneously after each sample rotation (Figs. 2 ▸ *b* and 2 ▸ *c*). Full pole figure coverage can be achieved by correct sample rotation (Fig. 2 ▸ *d*). Here, the χ axis of the sample rotation ring of the Euler cradle was set at an angle ω = 45° deviating from the incident-beam direction in the horizontal plane, and neutron diffractograms at sample orientations χ = 85, 65, 45, 25, 5° (for limestone, the measurements of χ = 5° neutron diffractograms were omitted owing to the limited beam time) and ϕ = 0, 15, 30,…, 345° were collected over the same proton pulse counts at the 2θ = 90° scattering detector bank. The *Q* range of the incident neutron beam in the single-frame mode was tuned to cover *Q* = 1.6–3.6 Å^−1^ (corresponding to a *d* range from 3.92 to 1.73 Å) to collect the 012 diffraction peak for texture measurement of the limestone sample. Another double-frame incident-beam mode with a wider *Q* range covering 1.3–12.5 Å^−1^ (corresponding to a *d* range from 4.7 to 0.5 Å) might be better to acquire more than 100 diffraction peaks, but the current incident neutron flux would be halved and consequently the collecting time would be ten times longer to get reliable statistics for the high-*Q* overlapping peaks around *Q* = 12.5 Å^−1^ (*d* = 0.5 Å).

Previously, partially dependent on the characteristic differences between neutron instruments, the shortest measurement time for each diffractogram (with a useable *Q* range covering 1.6–6.3 Å^−1^, *i.e.* a *d* range covering from 4.0 to 1.0 Å) of this limestone per sample rotation was 900 s on the HIPPO neutron diffractometer (Wenk *et al.*, 2003 ▸) with a neutron flux of 2.4 × 10^7^ n cm^−2^ s^−1^ at the sample scattering center (Matthies *et al.*, 2005 ▸). In a general texture measurement for such a sample, it took about 3.6 ks to collect 61 neutron diffraction peaks (with a *d* range from 4.0 to 0.9 Å) per sample rotation at the GPPD neutron diffractometer (Lutterotti *et al.*, 1997 ▸).

**Figure 3:**
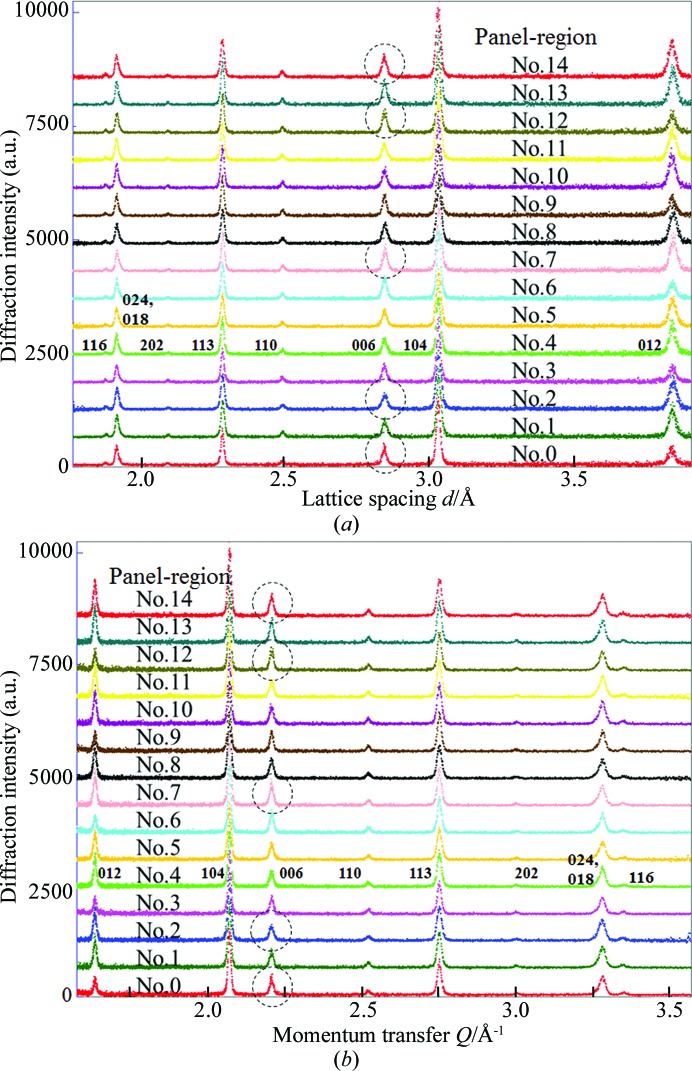
Examples of measured neutron diffractograms of the limestone reference sample, obtained from different panel regions at sample orientation angles of χ = 65° and ϕ = 0°. (*a*) Diffraction intensity *versus* lattice spacing *d*. (*b*) Diffraction intensity *versus* momentum transfer *Q*.

The limestone sample was rotated on its χ and ϕ axes in a way similar to conventional AD neutron diffraction texture measurement and only nine diffraction peaks were collected. The neutron diffractograms are shown in Fig. 3 ▸, plotted as diffraction intensity *versus* lattice spacing (*d*) and momentum transfer (*Q*). In the former diffractograms (Fig. 3 ▸ *a*), the local change in the peak intensities and shapes of diffraction peaks at larger *d* is more emphasized than the corrresponding change at smaller *d*. The latter diffractograms (Fig. 3 ▸ *b*) show a relative change in all the diffraction peaks in natural and linear reciprocal space and are very helpful for a global comparison of a particular orientation diffractogram of the textured sample with the corresponding random powder diffractogram. Here, a clear difference in diffraction intensities and peak shapes was found from the 006 reflections at sample orientation angles of χ = 65° and ϕ = 0° (as marked by dashed circles) when comparing the diffractograms from panel regions Nos. 0, 2, 7, 12 and 14. It is easy to understand that, from a neutron diffractogram collected from the whole south bank without any panel-region division, it is impossible to detect such a difference in these 006 reflection intensities among the various local panel regions.

The stereographic resolution of pole figure coverage (*i.e.* the angular resolving power in the pole figure, or the window by which the pole figure is scanned; Bunge *et al.*, 1982 ▸; Brokmeier *et al.*, 2011 ▸) is here simply represented by the No. 7 center panel region, at about Δχ = 4.0° and Δϕ = 5.5°. On the other hand, no panel-region division corresponds to a stereographic resolution of Δχ = 20.2° and Δϕ = 16.5°. It should be noted that the Δϕ part of the stereographic resolution is dependent on the inclination angle χ of the sample normal direction from the vertical *y* axis (Brokmeier *et al.*, 2011 ▸). Accordingly, all 15 panel regions were summarized into three bank groups with different diffraction angles 2θ = 100, 90, 80°. In other words, ‘high stereographic resolution’ here denotes a small stereographic angle span (for example, Δχ = 4.0° and Δϕ = 5.5° in pole figure coverage) to ensure an orientation-sensitive TOF neutron diffractogram, which is different from the ‘high ODF resolution’ at 5° mentioned below in relation to *MAUD* Rietveld texture analysis (Lutterotti *et al.*, 1997 ▸).

The pulsed proton beam involved in this study was operated at 25 Hz (25 pulses per second) with a proton beam power of around 200 kW, and 4.3 × 10^13^ neutrons were obtained at each proton pulse through the spallation reaction of mercury nuclei after collision between the proton beam and the mercury target vessel (Takada *et al.*, 2017 ▸). Consequently, the neutron flux at the sample scattering center of TAKUMI was about 9.6 × 10^6^ n cm^−2^ s^−1^ in the high-intensity experimental mode. The neutron collecting times per sample rotation were about 200 s (proton pulse counts: *T*_0_ = 5000), 40 s (*T*_0_ = 1000) and 30 s (*T*_0_ = 750) for limestone, multilayered steel and IF steel, respectively. The total measurement time for the limestone sample (96 rotations) was about 19.2 ks, and those for the multilayered steel and IF steel (120 rotations) were about 4.8 and 3.6 ks, respectively. Note that, for steels, the collecting time per sample rotation will be reduced in the near future to about 10 s when the J-PARC neutron target plan of about 1 MW proton beam power is achieved. The χ and ϕ sample rotations can be reduced by about 18 times by considering the sample symmetry of the sheet material and employing hexagonal equal-area pole figure coverage (Gnäupel-Herold & Creuziger, 2011 ▸) for diffractogram collection. In such a case, the use of a remote-controlled robot will be very valuable for sample exchange, alignment and rotation positioning.

A vanadium–nickel alloy powder sample packed into a vanadium sample can 10 mm in diameter and 64 mm in height was measured to correct the incident-beam intensity. Al_2_O_3_ and CeO_2_ powder samples packed into similar vanadium sample cans were employed to determine the instrumental parameters for Rietveld texture analysis of limestone and multilayered steel, respectively.

### 2.3. Neutron diffraction texture analysis   

The *Material Analysis Using Diffraction* (*MAUD*) software (Lutterotti *et al.*, 1997 ▸) was employed to analyze the bulk crystallographic texture on the basis of the extended Williams–Imhof–Matthies–Vinel (E-WIMV) method (Matthies & Vinel, 1982 ▸; Lutterotti *et al.*, 2004 ▸). For the limestone sample (CaCO_3_), the space group 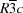, the lattice parameters *a* = 4.983 Å and *c* = 17.077 Å, and the *x*-axis fractional coordinate of the oxygen atom site *x*_0_ = 0.2558 from the literature (Lutterotti *et al.*, 1997 ▸) were employed as initial parameters for Rietveld analysis. Although the low number of diffraction peaks and weak peak intensities from the limestone texture measurement measured on TAKUMI may result in a less reliable Rietveld analysis (Lutterotti *et al.*, 1997 ▸), the crystal structure parameter, crystallite size and root-mean-square microstrain were still analyzed here as value-added information from the combined Rietveld texture and microstructure analysis.

The pole figures were recalculated from the above TAKUMI TOF diffractograms with no sample symmetry assumption at Rietveld-analysis ODF resolutions (*i.e.* ODF cell sizes; Matthies *et al.*, 2005 ▸) of 15 and 10°, and were then compared with the previously published texture results (Wenk *et al.*, 2003 ▸) obtained from established neutron beamlines, including the D20 high-intensity neutron diffractometer at Institut Laue–Langevin (ILL, Grenoble, France) with a one-dimensional PSD over a 2θ angular range of 120° and the HIPPO TOF neutron diffractometer with 2θ = 150, 90, 40° diffraction bank rings composed of 30 neutron detector panels. The count time on HIPPO was about 7.2 ks for eight sample rotations, while that on D20 was about 72 ks for a regular mesh in χ and ϕ of 10 × 10° and two ω positions (50 and 125°) (Wenk *et al.*, 2003 ▸; Vogel *et al.*, 2004 ▸). Given that the previously published D20 and HIPPO pole figures were plotted in a special manner (Wenk *et al.*, 2003 ▸; Vogel *et al.*, 2004 ▸) which it is not convenient to follow for the TAKUMI results, the limestone texture was measured again on HIPPO through eight sample rotations (ω = 0, 45, 67.5, 90, 112.5, 135, 157.5, 202.5°, with an ω offset of −61.7°, *i.e.* with the RD of the sample arrowed to the notch of the sample holder). The recalculated pole figures were analyzed to make a direct comparison, using about 110 diffraction peaks (*d* range ≃ 0.7–4.0 Å), of the HIPPO TOF neutron diffractograms from the first four sample rotations and from all eight sample rotations.

For the multilayered steel sample, as an example of a multiphase engineering material, the necessity of panel-region division for improving the texture precision was also investigated. More than eight diffraction peaks (*d* range 0.7–2.3 Å) were Rietveld analyzed for each constituent phase, and no sample symmetry was presumed for the pole figure recalculation. Multiphase textures with ODF resolutions of 10 and 5° were employed during the Rietveld texture analysis in four cases. Case I involved 1800 neutron diffractograms obtained from 120 sample rotations and panel-region division was employed at a high stereographic resolution of about Δχ = 4.0° and Δϕ = 5.5° in full pole figure coverage. Case II was at the same stereographic resolution as Case I, but only 525 neutron diffractograms were obtained from 35 rotations in about one-quarter pole figure coverage (*i.e.* χ = 85, 65, 45, 25, 5° and ϕ = 0, 15, 30,…, 90° were employed; Xu *et al.*, 2017 ▸). Case III was at the same stereographic resolution as Case I, but only 120 neutron diffractograms were employed through selecting the center panel region No. 7 neutron diffractograms, as shown in Fig. 2 ▸(*c*), from all 120 sample rotations. Case IV also used 120 neutron diffractograms obtained from 120 sample rotations but no panel-region division was employed, *i.e.* each neutron diffractogram was obtained with a low stereographic resolution of about Δχ = 20.2° and Δϕ = 16.5° in pole figure coverage through simply summing all the neutrons diffracted towards the 90° scattering detector bank. The multilayered steel sample was also measured on HIPPO using four sample rotations (ω = 0, 45, 67.5, 90°, total time 2.4 ks) to obtain 120 neutron diffractograms from the diffraction rings 2θ = 150, 90, 40°, and a *d* range of ∼0.7–2.3 Å and orthorhombic sample symmetry were employed to obtain analysis results that were as reliable as possible.

In a similar way to conventional AD neutron diffraction texture analysis, the 1800 neutron diffractograms from the multilayered steel were individually peak fitted to extract the martensite (110), (200), (211), (310) experimental pole figures and the austenite (111), (200), (220), (311) ones, and the corresponding orientation distribution functions (ODF_Bunge_) were calculated using the spherical-harmonic function series expansion method (Bunge, 1982 ▸).

### 2.4. Neutron diffraction macro stress tensor analysis   

The macro stress tensors {σ_11_, σ_22_, σ_33_, σ_13_, σ_23_, σ_12_} of the cold-rolled and annealed IF steel (11 is the rolling direction, 22 the transverse direction and 33 the normal direction) and the multilayered steel were analyzed. The former was employed to estimate the possible measurement error and the latter was employed to evaluate the phase stress tensors of austenite and martensite as a practical application example. Given the cubic shape of the steel samples, absorption effects were corrected for using the harmonic coefficient method (Wenk *et al.*, 2010 ▸).

The BulkPathGEO texture-weighted geometric mean micromechanical model implemented in the *MAUD* software was used here to take into account the influence of single-crystal elastic anisotropy and the bulk texture characteristics of polycrystalline metallic materials. The numerically exact inversion of the BulkPathGEO geometric mean model used for residual stress analysis may provide results that are closer to the self-consistent model than the results using the Voigt, Reuss, Hill stiffness and Hill compliance models (Lutterotti, 2010 ▸). The BulkPathGEO geometric mean model for stress analysis satisfies a physically grounded ‘inversion relation’, *i.e.* the bulk compliance, 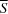, is the inversion of the bulk stiffness, 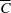, as follows (Matthies *et al.*, 2001 ▸; Lutterotti, 2010 ▸):
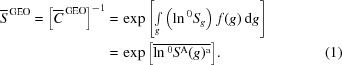
Here, *f*(*g*) is the ODF density at the grain orientation *g* with an orientation angle (φ_1_, Φ, φ_2_), and the superscript a denotes the arithmetic mean. The superscript A denotes the sample coordinate system. In addition, the diffraction elastic constants for each diffraction peak of the material were calculated from the single-crystal elastic constants 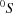, where the crystal properties were averaged using the moments of the ODF or pole figures, similar to those used for calculating bulk polycrystalline properties (Matthies *et al.*, 2001 ▸; Lutterotti, 2010 ▸). The above relation can be applied to correct the description of the strain/stress compatibility during the residual strain/stress analysis.

The single-crystal elastic constants of pure iron (*C*_11_ = 236.9 GPa, *C*_12_ = 140.6 GPa, *C*_44_ = 116.0 GPa; Wang *et al.*, 2003 ▸) were employed for the ferrite in the IF steel and the martensite in the multilayered steel, and those of austenite stainless steel (*C*_11_ = 198.0 GPa, *C*_12_ = 125.0 GPa, *C*_44_ = 122.0 GPa; Wroński *et al.*, 2007 ▸) were employed for the austenite in the multilayered steel. For these simple thin steel sheets, the plane-stress assumption was thought reasonable and the condition σ_33_ = σ_23_ = σ_13_ = 0 was employed to simplify the above macro stress calculation. Because there was no external macro stress loading for the multilayered steel, the composite macro stress tensor between austenite and martensite, σ_c,*ij*_ = (1 − *f*) σ_m,*ij*_ + *f*σ_a,*ij*_, was assumed here as 0, and the deviations of the corresponding composite stress tensors were used to estimate the measurement error of the residual macro stress. Here, the subscripts c, m and a denote composite, martensite and austenite, respectively, *ij* denotes 11, 22 or 12, and *f* is the volume fraction of martensite.

The strain pole figures of martensite and austenite were analyzed by individual peak fitting through taking the monolithic steel sheet as a stress-free reference roughly measured by RESA-2 angle-dispersive neutron diffraction, without distinguishing the macro strain pole figures from the intergranular micro strain pole figures. On the basis of the above analysis, the residual stress tensors of multilayered steel were evaluated quantitatively, and its formation mechanism is discussed below to reveal the significant informational role of combined Rietveld bulk texture and residual stress evaluation in research and development activities on advanced metallic materials.

## 3. Results and discussion   

### 3.1. Texture analysis of trigonal limestone   

**Figure 4:**
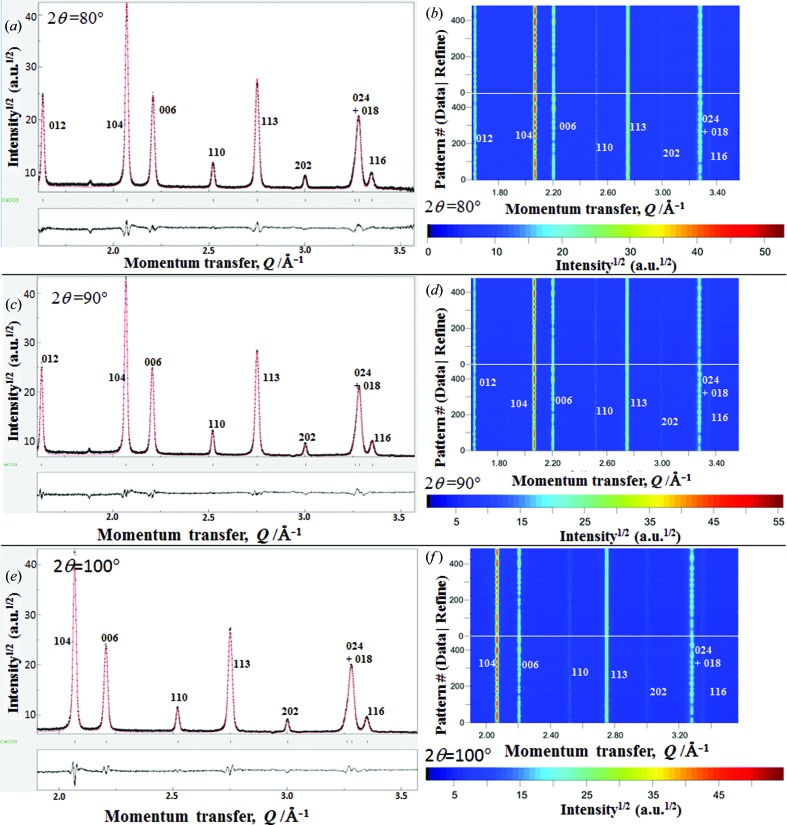
Combined *MAUD* Rietveld texture analysis of the limestone reference sample using all of the TOF neutron diffraction patterns collected at different nominal diffraction angles [(*a*), (*b*) 2θ = 80°; (*c*), (*d*) 2θ = 90°; (*e*), (*f*) 2θ = 100°] through panel-region divisions and sample rotations. (*a*), (*c*), (*e*) The averaged TOF neutron diffractograms, the Rietveld fitted patterns and their residual plots. (*b*), (*d*), (*f*) The two-dimensional mappings for the measured diffractograms (lower) and their Rietveld fitted profiles (upper).

Fig. 4 ▸ shows the combined Rietveld texture analysis of the limestone reference sample using all the TOF neutron diffractograms collected at different 2θ scattering angles. Although the averaged TOF neutron diffractograms (Figs. 4 ▸ *a*, 4 ▸ *c* and 4 ▸ *e*) look somewhat similar to each other, the differences in the residual plots between their refined profiles and the corresponding averaged TOF diffractograms prove that the averaged TOF diffractograms obtained from 480 sample orientations (through 96 sample rotations using the TOF neutron diffractograms from five different η panel regions with the same 2θ) are not completely random. On the other hand, the two-dimensional mappings for the measured TOF diffractograms from 2θ = 80, 90, 100° and the Rietveld refined two-dimensional patterns (Figs. 4 ▸ *b*, 4 ▸ *d* and 4*f*) show a satisfactory quality of Rietveld fit. The diffraction intensities and the shape broadening of reflections 012, 104 and 006 show relatively sharper changes between the different sample orientations and those of 113 show almost no change. This reveals that quite a high stereographic resolution has been achieved in this texture measurement mode.

**Figure 5:**
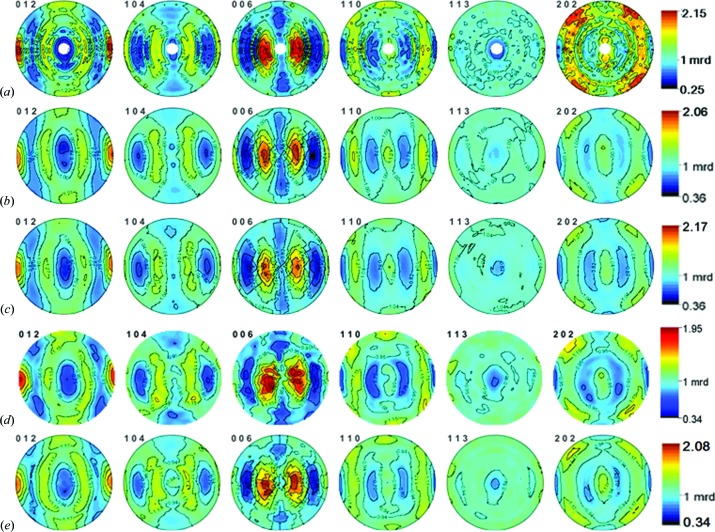
(*a*) Measured, (*b*) recalculated (ODF resolution = 15°) and (*c*) recalculated (ODF resolution = 10°) crystallographic orientation pole figures for the limestone reference sample in equal-area projection obtained from the TAKUMI TOF neutron diffractometer at J-PARC, compared with (*d*), (*e*) the recalculated pole figures (ODF resolution = 10°) from the HIPPO TOF neutron diffractometer at LANSCE with 2θ = 150, 90, 40° diffraction rings composed of different bank panels, (*d*) four sample rotations and (*e*) eight sample rotations.

The obtained lattice parameters of the trigonal crystal structure (with standard deviations obtained during the analysis given in parentheses) were *a* = 4.98235 (8) Å, *c* = 17.07758 (3) Å and *x*_0_ = 0.25428 (3), achieving a refinement index *R*_w_ = 0.102 (Lutterotti *et al.*, 1997 ▸; Chateigner, 2005 ▸) (*R*_w_ < 0.10 indicates a satisfactory powder diffraction structure analysis). The obtained crystallite size was 1512 (3) Å and the root-mean-square microstrain was 0.002604 (9), similar to the published results (Von Dreele, 1997 ▸; Lutterotti *et al.*, 1997 ▸; Wenk *et al.*, 2003 ▸). The six experimentally measured and Rietveld recalculated pole figures as required by the standard protocol (Fig. 5 ▸) are found to be comparable to the published recalculated pole figures (Wenk *et al.*, 2003 ▸) obtained from the D20 angle-dispersive neutron diffractometer with a one-dimensional PSD over a 2θ angular range of 120°, and from the HIPPO TOF neutron diffractometer with 2θ = 150, 90, 40° diffraction rings composed of different bank panels at LANSCE (Wenk *et al.*, 2003 ▸; Vogel *et al.*, 2004 ▸).

Since the integrated intensities of the 012 diffraction peaks collected from the panel regions with a centered 2θ of 100° were found to be much noisier than the other diffraction peaks (Figs. 4 ▸ *e* and 4 ▸ *f*), only eight diffraction peaks (*Q* range 1.89–3.55 Å^−1^ or *d* range from 3.30 to 1.76 Å) from these panel regions were employed in the Rietveld texture analysis, together with all nine diffraction peaks (*Q* range 1.59–3.55 Å^−1^ or *d* range from 3.91 to 1.76 Å) from the other panel regions with nominal 2θ = 80, 90°. The low statistics of the 012 diffraction peaks and backgrounds collected from the nominal 2θ = 100° were mostly related to the improper wavelength bandwidth setting. Here, our *d*_max_ at 2θ = 100° was less than 3.98 Å, and the 012 peak and its background were not fully observed. Such a phenomenon can be effectively avoided by using the double-frame medium-flux neutron beam mode with a wider *d* range.

Thanks to the crystallographic orientation information from the almost complete pole figure coverage, the uncertainty in the diffraction intensities of the diffraction peaks above 012 was balanced during Rietveld texture analysis. Actually, the measured (110) and (202) pole figures were less noisy than the previously published measured pole figures obtained using TOF neutron diffraction (Wenk, 1991 ▸; Walther *et al.*, 1995 ▸), suggesting that high stereographic resolution texture measurement after panel-region division is much more effective for evaluating various polycrystalline materials, from simple metallic alloys to complex geomaterials. It was found that the recalculated pole figures at an ODF resolution of 15° were consistent with the recently published recalculated pole figures from D20 and HIPPO, which represent two of the best data sets in the round-robin project (Wenk, 1991 ▸; Walther *et al.*, 1995 ▸; Wenk *et al.*, 2003 ▸; Von Dreele, 1997 ▸; Vogel *et al.*, 2004 ▸), substantiating the high reliability of the pole figures measured at TAKUMI. It seems that the results from D20 have been smoothed so that the maximum pole distribution density [1.87 multiples of a random distribution (m.r.d.)] is a little lower than all the results from the TOF method (*e.g.* 2.09 m.r.d. in the case of eight rotations on HIPPO) and the pole density distribution is not so clear around the maximum preferred orientation (Wenk, 1991 ▸; Vogel *et al.*, 2004 ▸). The recalculated pole figures at an ODF resolution of 10° from TAKUMI show the highest value in the maximum pole distribution density (2.17 m.r.d.) in the (006) pole figure with no evident ghost phenomenon. Moreover, a clearer symmetry of preferred orientations in the pole figures is found in Figs. 5 ▸(*b*) and 5 ▸(*c*) (obtained from TAKUMI by refining 8 peaks × 15 panel regions × 96 rotations + 1 peak × 10 panel regions × 96 rotations = 12 480 texture information units in 19.2 ks) than in Fig. 5 ▸(*d*) (obtained from HIPPO by refining 110 peaks × 30 panel regions × 4 rotations = 13 200 texture information units in 3.6 ks), suggesting that high stereographic resolution and appropriate rotations are very effective for improving texture precision. The increment in sample rotations on HIPPO from four times (Fig. 5 ▸ *d*) to eight times (Fig. 5 ▸ *e*) leads to an evident improvement in pole figure reliability.

For such texture measurement of a complex geomaterial sample, if the neutron collecting time after each sample rotation is extended enough to improve the data statistics of the diffraction peaks, and if the TOF binning width is reduced to increase the number of profile points for each diffractogram, a greater number of diffraction peaks in the low-*d* range may be effectively employed during combined Rietveld texture analysis and the sample rotations may be reduced (Wenk *et al.*, 2003 ▸; Von Dreele, 1997 ▸).

### 3.2. Texture analysis of multilayered steel   

Through Rietveld texture analysis using all 1800 neutron diffractograms after panel-region division during 120 sample rotations, the martensite in the multilayered steel was found to be 50.06 (3)% in volume fraction and the austenite 49.94 (3)%. Through Rietveld analysis of these 1800 high stereographic resolution neutron diffractograms from various sample orientations and considering the absorption correction, the lattice parameters of martensite and austenite were found to be 2.86679 (1) and 3.58357 (1) Å, respectively, revealing a much higher statistical accuracy of 10^−6^ than the conventional 10^−5^ statistical accuracy of simple residual stress measurement. The coherent crystallite sizes of martensite and austenite analyzed from peak-shape broadening were 1068 (5) and 1796 (3) Å, respectively. The root-mean-square microstrains analyzed from peak-shape broadening were 3450 (3) × 10^−6^ for as-quenched martensite layers with residual transformation (elastoplastic) strain, and 1020 (3) × 10^−6^ for non-transformed austenite layers only accompanied by thermal (elastic) strain. Such results are reasonable for the microstructure characteristics of martensite and austenite in multilayered steel, although further quantitative examination is necessary.

**Figure 6:**
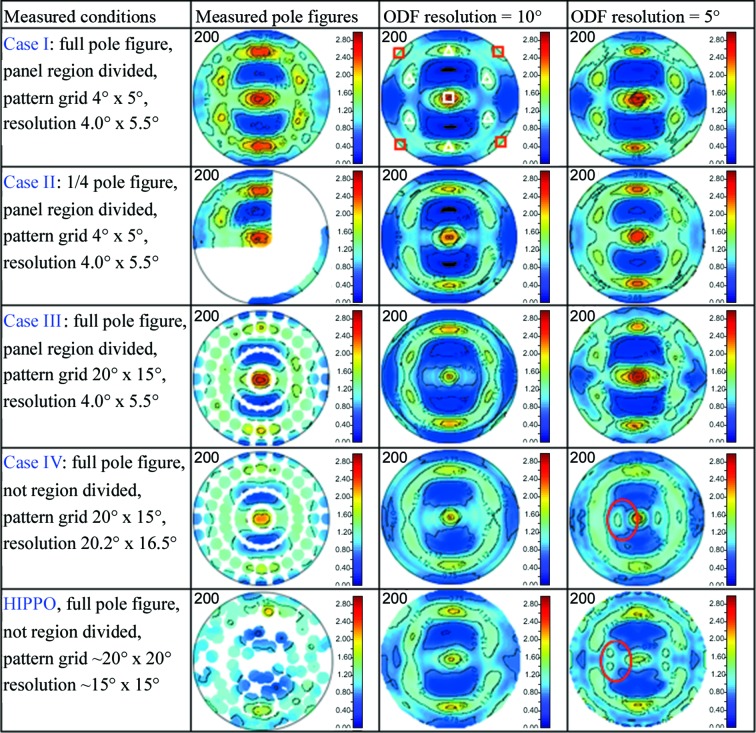
Martensite (200) crystallographic orientation pole figures of the multilayered steel (*d* range ∼0.7–2.3 Å) in equal-area projection. Here, the pattern grid is the misorientation in stereographic angle (χ, ϕ) between neighboring neutron diffractograms, and the resolution is the stereographic resolution to obtain a neutron diffractogram. The ODF resolution is the ODF cell size during Rietveld pole figure analysis. White open triangles denote {111}〈112〉 components and red open squares denote {100}〈011〉 components.

Fig. 6 ▸ shows the measured and reconstructed martensite (200) pole figures based on the TAKUMI neutron diffractograms under various analysis conditions, together with those from HIPPO. Case I led to a smooth and strong martensite (200) pole figure distribution at an ODF resolution of 5°, while the corresponding (200) pole figure with an ODF resolution of 10° was a little weak in its maximum distribution intensity. Case II led to a (200) pole figure consistent with that of Case I at an ODF resolution of 5°, and the existence of surplus data revealed that the 18 sample rotations in the hexagonal equal-area pole figure coverage (Gnäupel-Herold & Creuziger, 2011 ▸) should also be suitable for measuring the texture of a sheet material. By optimizing the ω angle of the Eulerian cradle to make better use of all the 2θ = ±90° neutron detector banks, the sample rotation time may be further reduced. Case III used the same panel-region division, and the 120 uniformly distributed neutron diffractograms resulted in a strong but not so smooth martensite (200) pole figure with an ODF resolution of 5°, while the maximum intensity with an ODF resolution of 10° was also a little weak owing to orientation averaging with the neighboring orientations. Case IV did not use panel-region division and led to a clearly weakened texture. The finer orientation cells at an ODF resolution of 10° in the E-WIMV texture model cannot precisely evaluate the real texture, and the limited number of neutron diffractograms (*i.e.* limited amount of texture information) led to some ghost distribution around the central {001}〈110〉 component at an ODF resolution of 5°, marked by a red oval. In comparison, the fine panel-region division improved the stereographic resolution to Δχ = 4.0° and Δϕ = 5.5°, and use of the same number of neutron diffractograms as in Case III may improve the precision of the texture evaluation in Case IV. For the recalculated pole figures from HIPPO with an assumption of orthorhombic sample symmetry, no ghost preferred orientation was found at an ODF resolution of 10°, but the maximum pole density was a little less than those from Cases I–III; at an ODF resolution of 5°, some ghost distribution appeared, similar to Case IV.

From the above comparisons, it can be seen that the ‘high stereographic resolution’ orientation-sensitive TOF neutron diffractograms obtained from fine panel-region division during texture measurement enable us to easily achieve ‘high ODF resolution’ Rietveld texture analysis. The pole figure comparison in Fig. 6 ▸ confirms that high stereographic resolution neutron diffractograms are very efficient for enhancing the texture reliability of reconstructed pole figures, and fine changes in averaged crystal rotation and texture evolution can be evaluated reliably. This is very valuable for research topics related to low cycle fatigue, creep failure, hydrogen embrittlement, thermal stress relaxation and so on. On the other hand, too fine a panel-region division may decrease the counting statistics of the diffraction intensities so that a longer neutron counting time would be required for reliable Rietveld texture analysis, which is not welcome during high-temperature deformation and/or microstructure evolution experiments. In addition, it is clear that Rietveld texture analysis using a large number of neutron diffractograms may delay the analysis process, so optimization of the sample rotation and panel-region division is necessary, and the simplest way might be to measure the texture of a sheet material by considering the sample symmetry.

**Figure 7:**
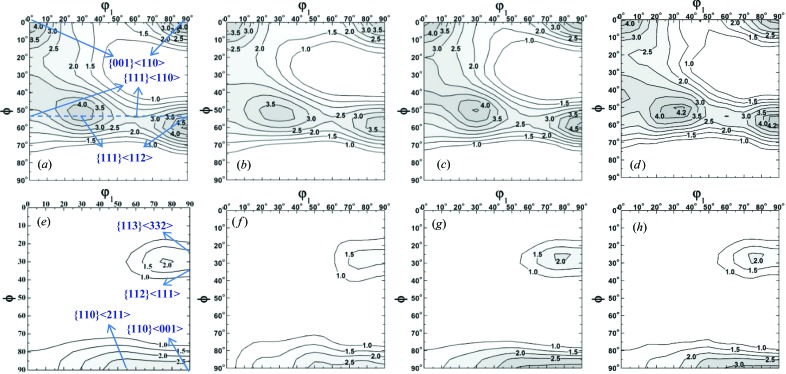
φ_2_ = 45° ODF_Bunge_ sections of (*a*)–(*d*) martensite and (*e*)–(*h*) austenite textures in the multilayered steel sample obtained at an ODF resolution of 5°. (*a*), (*e*) RESA-2 AD neutron diffraction with a one-dimensional PSD (Xu *et al.*, 2012 ▸) and individual peak fitting. (*b*), (*f*) TAKUMI TOF neutron diffraction (1800 diffractograms), individual peak fitting, pattern grid 4 × 5°, panel-region stereographic resolution 4.0 × 5.5°. (*c*), (*g*) TAKUMI TOF neutron diffraction (1800 diffractograms, *i.e.* Case I), Rietveld texture analysis, pattern grid 4 × 5°, panel-region stereographic resolution 4.0 × 5.5°. (*d*), (*h*) TAKUMI TOF neutron diffraction (525 diffractograms, *i.e.* Case II), Rietveld texture analysis, pattern grid 4 × 5°, panel-region stereographic resolution 4.0 × 5.5°.

From the recalculated pole figures using a Rietveld analysis ODF resolution of 5° obtained from the Rietveld texture analysis of TAKUMI neutron diffractograms, new orientation distribution functions (ODF_Bunge_) using Bunge’s harmonic series expansion method (*L*_max_ = 32) were calculated in order to compare the martensite bulk textures quantitatively. The ODF_Bunge_ in Fig. 7 ▸(*b*) was calculated from the integrated intensities of the martensite 110, 200 and 211 reflections extracted from neutron diffractograms using the individual peak-fitting method. It shows a slightly weaker texture than that from Rietveld texture analysis (Fig. 7 ▸ *c*), confirming that Rietveld texture analysis involving more reflections may improve the texture reliability to a certain extent. Comparison of the φ_2_ = 45° ODF_Bunge_ sections from RESA-2 individual peak fitting (Fig. 7 ▸ *a*) and TAKUMI individual peak fitting (Fig. 7 ▸ *b*) suggests that the relatively low stereographic resolution on TAKUMI cannot ensure a high-precision texture analysis by the individual fitting of a few diffraction peaks. On the other hand, for Rietveld texture analysis, the φ_2_ = 45° ODF section (Fig. 7 ▸ *d*) using 525 diffractograms shows a quite similar ODF_Bunge_ result to those using 1800 diffractograms (Fig. 7 ▸ *c*). These comparisons suggest that Rietveld texture analysis over a wide *d* range during TOF neutron diffraction texture evaluation ensures high-accuracy ODF data as good as or better than conventional angle-dispersive neutron diffraction texture evaluation at a high orientation resolution. These precise pole figures and the φ_2_ = 45° ODF_Bunge_ sections of the martensite show typical cold-rolled ferrite/martensite texture characteristics, suggesting that a strong texture memory effect has occurred in the martensite layers during rapid solution treatment. Those of the austenite show typical recrystallized texture characteristics, which is consistent with the equiaxial morphology of austenite grains shown in Fig. 1 ▸(*b*), suggesting that full recrystallization of cold-rolled austenite occurred during the heating and short 1273 K isothermal holding period of rapid solution treatment and was then stably retained to room temperature without evident martensite transformation.

### 3.3. Macro stress tensor analysis of IF steel and multilayered steel   

The macro stress tensors {σ_11_, σ_22_, σ_33_, σ_13_, σ_23_, σ_12_} of the IF steel sheet with and without a plane stress assumption σ_13_ = σ_23_ = σ_33_ = 0 were calculated using the BulkPathGEO texture-weighted geometric mean model and the texture ODF as measured in Case I. They were {26.3 (4), 10.0 (3), 0, 0, 0, −0.1 (2)} MPa and {26.7 (7), 9.9 (3), −9.8 (1), 4.2 (3), 23.7 (6), −0.1 (4)} MPa, respectively, suggesting that the residual macro stress in the IF steel sheet was almost removed through the annealing treatment. Given that no external loading existed for this 15 × 15 × 15 mm cubic sample and the steel sheets were annealed, in addition to the recrystallization texture characteristics and the equiaxial grain morphology (Xu *et al.*, 2008 ▸), the above nonzero stress tensor components were here accepted as the measurement error, and the plane stress assumption was deemed reasonable. On the other hand, using the WSODF texture-weighted strain ODF model (Popa & Balzar, 2001 ▸; Balzar *et al.*, 2010 ▸), the recalculated strain pole figures of the IF steel sheet had a macro strain deviation of about ±130 × 10^−6^ from the ideal strain-free tensor, corresponding to a stress precision of about ±26 MPa. Consequently, the 10 × 10 × 10 mm cubic sample of multilayered steel was expected to have a similar or smaller measurement error.

The macro stress tensors of the martensite and austenite in the multilayered steel under a plane stress assumption are shown in Table 1 ▸, calculated using the BulkPathGEO geometric mean model and the E-WIMV modeled textures of the martensite and austenite. The standard deviation of the macro stress tensor components is within ±5 MPa, revealing that the precision is satisfactory. According to the composite stress law, the ideal composite stress should be zero and the actual composite stress was employed here to estimate the stress measurement error. For Cases I–IV, the stress component balance within ±20 MPa for the multilayered steel was consistent with that for the IF steel. Given that the wide beam size and the variation in the diffraction gauge volume center during the orientation rotation of the cubic sample may result in a large stress measurement error (Suzuki *et al.*, 2013 ▸), a neutron diffraction experimental investigation and related theoretical study will be very valuable to clarify the effects of sample size and shape on the accuracy of the macro stress tensor and further optimize the absorption correction (Wenk *et al.*, 2010 ▸).

When the multiphase textures and the macro stresses were refined simultaneously, the macro stress tensor obtained using the BulkPathGEO model was at a stress level of 320 MPa, and there was a trend that the macro stress tensor at low stereographic resolution resulted in a higher macro stress level (Case IV, about 340 MPa) than that at a high stereographic resolution (Case I, about 320 MPa; Case III, about 330 MPa). This suggests that high-resolution texture measurement is valuable for a precise stress evaluation (Matthies *et al.*, 2001 ▸), especially when the elastic anisotropy of the single crystal under investigation (*e.g.* Au, Mg, Zn and/or their alloys) is more evident and/or the bulk averaged texture is sharper. Here, because the elastic anisotropy for single-crystal iron is not large (Matthies *et al.*, 2001 ▸) and the textures of austenite and martensite are not so sharp, the obtained macro stresses with and without taking the elastic anisotropy into account (Case I *versus* Case I*a*) had no evident change. However, if the texture effect is not completely considered during Rietveld macro stress tensor analysis, the residual of the refined profiles will be much larger and so the corresponding stress tensor will obviously deviate from the stress tensors shown in Table 1 ▸.

These macro stress tensors revealed an RD–TD in-plane compressive stress distribution in the martensite layers and an RD–TD in-plane tensile stress distribution in the austenite layers. Such phase stress partitioning occurs for two reasons: (i) the approximate Δ*V*/*V* = 3.5% volume expansion of the martensite transformation (Moyer & Ansell, 1975 ▸; Ray *et al.*, 1994 ▸) during the water quenching of the martensite layers containing 0.32 C mass%; and (ii) the difference in the linear expansion coefficients between the SUS301 austenite (α_a_ = 16.9 × 10^−6^ K^−1^) and the SUS420J2 martensite (α_m_ = 10.3 × 10^−6^ K^−1^) (Nippon Steels & Sumitomo Metals, 2017 ▸). Here, relative to the austenite layers, the volume expansion from the martensite transformation may lead to (Δ*L*/*L*)_transformation_ = 1/3 Δ*V*/*V* = 1/3 × 3.5% = 1.16% relative linear expansion in the martensite layers, and the difference in the linear expansion coefficients between the austenite and the martensite from Ms ≃ 798 K to room temperature (298 K) may lead to about (Δ*L*/*L*)_thermal_ = (α_a_ − α_m_)Δ*T* = (16.9 − 10.3) × 10^−6^ × (798 − 298) = 0.33% relative linear expansion in martensite. Therefore, if the compositional gradient-induced strain relaxation across the martensite/austenite interlayers is omitted, about a 1.5% linear expansion misfit in the RD–TD plane of the martensite layers relative to that of the austenite layers will appear at room temperature, finally resulting in RD–TD in-plane compressive stress in the martensite layers and RD–TD in-plane tensile stress in the austenite layers.

Considering that the martensite transformation is involved in the formation of martensite substructures consisting of high-density dislocations and/or twins and given that it depends on the applied hydro­static pressure (Moyer & Ansell, 1975 ▸; Ray *et al.*, 1994 ▸), it is reasonable to attribute the above stress partitioning mainly to the anisotropic expansion of the new martensite unit cell (*a*_m_, *a*_m_, *c*_m_) from the previous austenite unit cell (*a*_a_/2^1/2^, *a*_a_/2^1/2^, *a*_a_) according to the Bain (or simplified Kurdjumow–Sachs) orientation relationship (Ray *et al.*, 1994 ▸). Because the compressive strain occurring in ferrite during the diffusional ferrite transformation is related to the texture memory effect between the initial ferrite and the new ferrite after the ferrite→austenite→ferrite heating–cooling process (Xu *et al.*, 2013 ▸), the RD–TD in-plane compressive strain in the martensite should have a direct influence on the texture evolution during the martensite phase transformation.

**Figure 8:**
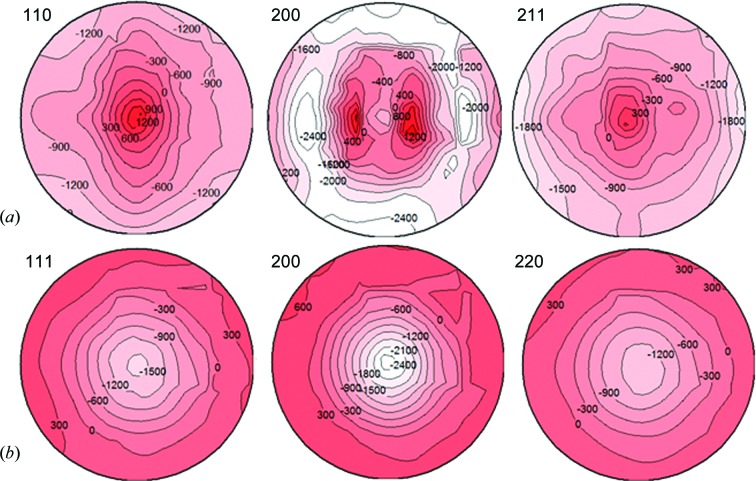
Experimental strain pole figures for (*a*) martensite and (*b*) austenite in the multilayered steel sample using the individual peak-fitting method (in equal-area projection, contour scale ×10^−6^), where *d*_0_ refers to the averaged lattice constant from the corresponding monolithic steel sheets after annealing at 973 K for 3.6 ks.

Fig. 8 ▸ shows the measured strain pole figures from individual peak fitting of TAKUMI TOF diffractograms, referring to the stress-free lattice parameters of the martensite and austenite monolithic steel sheets measured on RESA-2. Note that the stress-free lattice plane spacings *d*_0_ of the martensite and austenite were not very precise because of the limited sample rotations, so only the trend of strain pole distribution should be considered here. For the austenite, the pole distributions of the RD–TD in-plane tensile strain and ND out-of-plane compressive strain were consistent with the above macro stress analysis results, because no transformation strain occurred in the final SUS301 austenite layers except strain partitioning due to the martensite transformation expansion and the subsequent thermal contraction misfit of the martensite layers newly transformed from the high-temperature austenite. For the martensite, the pole distributions of the RD–TD in-plane compressive strain and ND out-of-plane tensile strain were very complex, especially in the (200) strain pole figure, because they were overlapped with the intergranular strains related to the variant selection of the martensite transformation. Therefore, in order to discuss the pole distribution of the martensite strain, it is necessary to separate the macro strain pole figures from the measured strain pole figures using the WSODF model by changing the expansion series of the texture-weighted strain/stress generalized spherical function (Popa & Balzar, 2001 ▸; Balzar *et al.*, 2010 ▸). The results are valuable for clarifying the effect of the transformation strain field on the orientation variant selection during the martensite transformation and clarifying the texture memory effect between the as-received initial martensite and the newly transformed martensite after rapid solution heating and quenching. Given the complexity of the related results and discussion, they will be the focus of another paper.

## 4. Conclusions   

Bulk textures and residual stress tensors of advanced multiphase structural materials are regarded as very valuable for investigating microstructure evolution, elastic anisotropy, elastoplastic deformation, delayed fracture, low cycle fatigue behavior and so on. Precise texture measurement using neutron diffraction is essential to carry out reliable macro stress tensor evaluation, even though an uncertain shear strain field exists in these textured materials. In this paper, an approach for combined analysis of high stereographic resolution texture and residual stress was established for the TAKUMI engineering materials neutron diffractometer through appropriate division of the neutron panel regions. The pole figure evaluation results of an experimentally deformed limestone standard sample with a trigonal crystal structure suggested that the obtained texture measurement precision is comparable to that of established neutron beamlines utilized for texture measurement, such as the HIPPO diffractometer at LANSCE and the D20 angle-dispersive neutron diffractometer at ILL. A high-strength martensite–austenite multilayered steel was employed as an example of a multiphase material to examine the reliability of simultaneous Rietveld analysis of the textures and stresses. Using the BulkPathGEO geometric mean micromechanical model implemented in the *MAUD* software, the macro stress tensor analysis with a plane stress assumption confirmed an RD–TD in-plane compressive stress distribution (about −330 MPa) in the martensite layers and an RD–TD in-plane tensile stress distribution (about 320 MPa) in the austenite layers. Such a phase stress partitioning distribution is mainly due to the additive effect of the volume expansion during martensite transformation and the linear contraction misfit between the austenite layers and the newly transformed martensite layers during water quenching in the sample preparation process.

## Figures and Tables

**Table 1:** Macro stress tensors of martensite and austenite in the multilayered steel sample under the plane stress assumption (σ_33_ = σ_23_ = σ_13_ = 0)

Stress analysis conditions	Phase	σ_11_ (MPa)	σ_22_ (MPa)	σ_12_ (MPa)
Case I. Full pole figure, pattern grid 4 × 5°, panel-region stereographic resolution 4.0 × 5.5°	Martensite	−327 (1)	−334 (1)	−5.0 (7)
	Austenite	312 (1)	321 (1)	2.6 (2)
	Composite stress	−8 (1)	−7 (1)	
Case I*a*. The same as Case I, but texture ODF-induced elastic anisotropy was not taken into consideration during BulkPathGEO analysis	Martensite	−341 (1)	−356 (1)	0.2 (7)
	Austenite	317 (1)	316 (1)	−2.1 (2)
	Composite stress	−12 (1)	−20 (1)	
Case II. Quarter pole figure, pattern grid 4 × 5°, panel-region orientation resolution 4.0 × 5.5°	Martensite	−334 (3)	−348 (3)	27.5 (4)
	Austenite	298 (2)	312 (3)	11.0 (1)
	Composite stress	−18 (3)	−18 (3)	
Case III. Full pole figure, pattern grid 20 × 15°, panel-region stereographic resolution 4.0 × 5.5°	Martensite	−316 (4)	−320 (4)	5.6 (2)
	Austenite	356 (2)	344 (2)	−2.0 (2)
	Composite stress	−20 (3)	12 (3)	
Case IV. Full pole figure, pattern grid 20 × 15°, panel-region stereographic resolution 20.2 × 16.5°	Martensite	−364 (5)	−355 (5)	2 (2)
	Austenite	330 (2)	319 (2)	−1.1 (8)
	Composite stress	−17 (4)	−18 (4)	

## References

[bb1] Balzar, D., Popa, N. C. & Vogel, S. C. (2010). *Mater. Sci. Eng. A*, **528**, 122–126.

[bb2] Brokmeier, H. G., Gan, W. M., Randau, C., Völler, M., Rebelo-Kornmeier, J. & Hofmann, M. (2011). *Nucl. Instrum. Methods Phys. Res. A*, **642**, 87–92.

[bb3] Bunge, H. J. (1982). *Texture Analysis in Materials Science*. London: Butterworths.

[bb4] Bunge, H. J., Wenk, H. R. & Pannetier, J. (1982). *Textures Microstruct.* **5**, 153–170.

[bb5] Chateigner, D. (2005). *J. Appl. Cryst.* **38**, 603–611.

[bb6] Coelho, R. S., Klaus, M. & Genzel, Ch. (2010). *J. Appl. Cryst.* **43**, 1322–1328.

[bb7] Conrad, H., Brückel, T., Schäfer, W. & Voigt, J. (2008). *J. Appl. Cryst.* **41**, 836–845.

[bb8] Ferrari, M. & Lutterotti, L. (1994). *J. Appl. Phys.* **76**, 7246–7255.

[bb9] Fitzpatrick, M. E., Fry, A. T., Holdway, P., Kandil, F. A., Shackleton, J. & Suominen, L. (2005). *Measurement Good Practice*, Guide No. 52, *Determination of Residual Stresses by X-ray Diffraction*, Issue 2, pp. 6–10. London: National Physical Laboratory.

[bb10] Gnäupel-Herold, T. & Creuziger, A. (2011). *Mater. Sci. Eng. A*, **528**, 3594–3600.

[bb11] Hansen, T. C., Henry, P. F., Fischer, H. E., Torregrossa, J. & Convert, P. (2008). *Meas. Sci. Technol.* **19**, 034001.

[bb12] Harjo, S., Ito, T., Aizawa, K., Arima, H., Abe, J., Moriai, A., Iwahashi, T. & Kamiyama, T. (2011). *Mater. Sci. Forum*, **681**, 443–448.

[bb13] Kockelmann, W., Chapon, L. C. & Radaelli, P. G. (2006). *Physica B*, **385–386**, 639–643.

[bb14] Krawitz, A. D. (2001). *Introduction to Diffraction in Materials Science and Engineering.* New York: John Wiley and Sons.

[bb15] Larsson, C., Clausen, B., Holden, T. M. & Bourke, M. A. M. (2004). *Scr. Mater.* **51**, 571–575.

[bb16] Li, M. J., Liu, X. L., Liu, Y. T., Zhang, M. Y., Wang, C. & Chen, D. F. (2016). *Acta Metall. Sin.* **52**, 463–472.

[bb17] Liss, K. D., Bartels, A., Schreyer, A. & Clemens, H. (2003). *Textures Microstruct.* **35**, 219–252.

[bb18] Lutterotti, L. (2010). *Nucl. Instrum. Methods Phys. Res. B*, **268**, 334–340.

[bb19] Lutterotti, L., Chateigner, D., Ferrari, S. & Ricote, J. (2004). *Thin Solid Films*, **450**, 34–41.

[bb20] Lutterotti, L., Matthies, S., Wenk, H. R., Schultz, A. J. & Richardson, J. (1997). *J. Appl. Phys.* **81**, 594–600.

[bb21] Mamun, A. A., Moat, R. J., Kelleher, J. & Bouchard, P. J. (2014). *Mater. High Temp.* **31**, 378–382.

[bb22] Matthies, S., Pehl, J., Wenk, H.-R., Lutterotti, L. & Vogel, S. C. (2005). *J. Appl. Cryst.* **38**, 462–475.

[bb23] Matthies, S., Priesmeyer, H. G. & Daymond, M. R. (2001). *J. Appl. Cryst.* **34**, 585–601.

[bb24] Matthies, S. & Vinel, G. W. (1982). *Phys. Status Solidi B*, **112**, K111–K114.

[bb25] Moriai, A., Torii, S., Suzuki, H., Harjo, S., Morii, Y., Arai, M., Tomota, Y., Suzuki, T., Akiniwa, Y., Kimura, H. & Akita, K. (2006). *Physica B*, **385–386**, 1043–1045.

[bb26] Moyer, J. M. & Ansell, G. S. (1975). *Metall. Trans. A*, **6**, 1785–1791.

[bb27] Nakajima, K. *et al.* (2017). *Quantum Beam Sci.* **1**, 9. https://doi.org/10.3390/qubs1030009.

[bb28] Nippon Steels & Sumitomo Metals (2017).* Steel Types, Mechanical Properties and Their Application to Stainless Steels*. Nippon Steels & Sumitomo Metals Co. Ltd, Tokyo, Japan. http://nssc.nssmc.com/pdf/product/stainless_sheet_j_13-03.pdf.

[bb29] Onuki, Y., Hoshikawa, A., Sato, S., Xu, P. G., Ishigaki, T., Saito, Y., Todoroki, H. & Hayashi, M. (2016). *J. Appl. Cryst.* **49**, 1579–1584.

[bb30] Popa, N. C. & Balzar, D. (2001). *J. Appl. Cryst.* **34**, 187–195.

[bb31] Ray, R. K., Jonas, J. J. & Hook, R. E. (1994). *Int. Mater. Rev.* **39**, 129–172.

[bb32] Rietveld, H. M. (1969). *J. Appl. Cryst.* **2**, 65–71.

[bb33] Sakasai, K. *et al.* (2017). *Quantum Beam Sci.* **1**, 10. https://doi.org/10.3390/qubs1020010.

[bb34] Simons, H., Daniels, J. E., Studer, A. J., Jones, J. L. & Hoffman, M. (2014). *J. Electroceram.* **32**, 283–291.

[bb35] Suzuki, H., Harjo, S., Abe, J., Xu, P. G., Aizawa, K. & Akita, K. (2013). *Nucl. Instrum. Methods Phys. Res. A*, **715**, 28–38.

[bb36] Suzuki, T., Yamanaka, K., Ishino, M., Shinohara, Y., Nagai, K., Tsuru, E. & Xu, P. G. (2012). *Tetsu Hagane*, **98**, 262–266.

[bb37] Takada, H., Haga, K., Teshigawara, M., Aso, T., Meigo, S., Kogawa, H., Naoe, T., Wakui, T., Ooi, M., Harada, M. & Futakawa, M. (2017). *Quantum Beam Sci.* **1**, 8. https://doi.org/10.3390/qubs1020008.

[bb38] Vogel, S. C., Hartig, C., Lutterotti, L., Von Dreele, R. B., Wenk, H. R. & Williams, D. J. (2004). *Adv. X-ray Anal.* **47**, 431–436.

[bb39] Von Dreele, R. B. (1997). *J. Appl. Cryst.* **30**, 517–525.

[bb40] Walther, K., Heinitz, J., Ullemeyer, K., Betzl, M. & Wenk, H.-R. (1995). *J. Appl. Cryst.* **28**, 503–507.

[bb41] Wang, X.-L., Wang, Y. D. & Richardson, J. W. (2002). *J. Appl. Cryst.* **35**, 533–537.

[bb42] Wang, Y. D., Wang, X.-L., Stoica, A. D., Richardson, J. W. & Lin Peng, R. (2003). *J. Appl. Cryst.* **36**, 14–22.

[bb43] Wenk, H.-R. (1991). *J. Appl. Cryst.* **24**, 920–927.

[bb45] Wenk, H. R., Lutterotti, L., Kaercher, P., Kanitpanyacharoen, W., Miyagi, L. & Vasin, R. (2014). *Powder Diffr.* **29**, 220–232.

[bb46] Wenk, H. R., Lutterotti, L. & Vogel, S. C. (2003). *Nucl. Instrum. Methods Phys. Res. A*, **515**, 575–588.

[bb47] Wenk, H. R., Lutterotti, L. & Vogel, S. C. (2010). *Powder Diffr.* **25**, 283–296.

[bb44] Wenk, H. R. & Van Houtte, P. (2004). *Rep. Prog. Phys.* **67**, 1367–1428.

[bb48] Wenk, H. R., Vasin, R. N., Kern, H., Matthies, S., Vogel, S. C. & Ivankina, T. I. (2012). *Tectonophysics*, **570–571**, 123–134.

[bb49] Wroński, S., Baczmański, A., Dakhlaoui, R., Braham, C., Wierzbanowski, K. & Oliver, E. C. (2007). *Acta Mater.* **55**, 6219–6233.

[bb50] Xu, P. G., Akita, K., Suzuki, H., Metoki, N. & Moriai, A. (2012). *Mater. Trans.* **53**, 1831–1836.

[bb51] Xu, P. G., Tomota, Y., Arakaki, Y., Harjo, S. & Sueyoshi, H. (2017). *Mater. Charact.* **127**, 104–110.

[bb52] Xu, P. G., Tomota, Y., Suzuki, H., Suzuki, T., Machiya, S. & Yin, F. X. (2008). *Mater. Trans.* **49**, 2033–2039.

[bb53] Xu, P. G., Tomota, Y., Vogel, S. C., Suzuki, T., Yonemura, M. & Kamiyama, T. (2013). *Rev. Adv. Mater. Sci.* **33**, 389–395.

[bb54] Xu, P. G., Yin, J. & Zhang, S. Y. (2015). *Acta Metall. Sin.* **51**, 1297–1305.

[bb55] Zhang, J., Kisi, E. H. & Kirstein, O. (2011). *J. Appl. Cryst.* **44**, 1062–1070.

